# Does adult attachment mediate the relationship between primary emotion traits and eating disorder symptoms?

**DOI:** 10.3389/fpsyg.2024.1372756

**Published:** 2024-05-02

**Authors:** Lisa Roithmeier, Jürgen Fuchshuber, Theresa Prandstätter, Deborah Andres, Beate Schmautz, Andreas Schwerdtfeger, Human-Friedrich Unterrainer

**Affiliations:** ^1^Institute of Psychology, University of Graz, Graz, Austria; ^2^University Clinic of Psychiatry and Psychotherapeutic Medicine, Medical University Graz, Graz, Austria; ^3^Center for Integrative Addiction Research (CIAR), Grüner Kreis Society, Vienna, Austria; ^4^Department of Psychoanalysis and Psychotherapy, Medical University Vienna, Vienna, Austria; ^5^Comprehensive Center for Clinical Neurosciences and Mental Health, Medical University Vienna, Vienna, Austria; ^6^Department of Religious Studies, University of Vienna, Vienna, Austria; ^7^Faculty of Psychotherapy Science, Sigmund Freud Private University, Vienna, Austria

**Keywords:** primary emotions, attachment, attachment anxiety, eating disorders, personality, eating behavior

## Abstract

**Objectives:**

Primary emotion traits and attachment patterns, have been linked to various mental disorders. This study aims to shed more light on the less studied relationship with eating disorder (ED) symptoms.

**Methods:**

A total of 921 non-clinical subjects (69.9% females) were assessed for primary emotions traits (B-ANPS-GL), attachment insecurity (ECR-RD8), and eating disorder pathology (EDE-Q8). A theoretically derived model was evaluated by means of a path analysis with attachment anxiety as assumed mediator variable.

**Results:**

Global problematic eating behavior showed negative correlations with the positive emotions PLAY, CARE, and LUST (*r* = −0.10 to −0.24), positive correlations with the negative primary emotions ANGER, FEAR, and SADNESS (*r* = 0.12–0.27), as well as with attachment anxiety (*r* = 0.22, all *p* < 0.01). Path analyses revealed direct effects between eating behavior pathology with LUST (β = −0.07 to −0.15) and FEAR (β = 0.12–0.19; all *p* < 0.05). The association of SADNESS and Weight (β = 0.05) and Shape Concern (β = 0.06, *p* < 0.001) was fully mediated by attachment anxiety. Overall, the path model explained 17% of the variance for attachment anxiety and 6% of the Restraint, 13% for Eating, 10% for Weight and 14% for Shape Concern Subscales.

**Discussion:**

The findings shed light on the multifactorial relationship between affective traits, attachment security, and eating disorder pathology. In line with previous research, the results emphasize the role of attachment and affective functioning in ED symptoms.

## 1 Introduction

Eating disorders (ED), such as anorexia or bulimia nervosa and binge eating disorder, are relatively common syndromes with a high rate of comorbidities and health risks. In Europe, occurrence rates of up to 4% were observed for female population, while in men this is significantly lower at around 1%, and yet only about one third of these are recognized as such by the healthcare system (Keski-Rahkonen and Mustelin, 2016). Treasure et al. (2020, p. 899) referred to eating disorders as “disabling, deadly, and costly mental disorders that considerably impair physical health and disrupt psychosocial functioning.”

Regarding the affective underpinnings of EDs the concept of primary emotions traits – developed by Jaak Panksepp – might be an interesting framework for etiological explorations.

As outlined in Panksepp’s (2004) seminal work on biologically shared mammalian affective systems, the proposed primary emotions can be roughly divided based on the respective valence. He assumed four positive or pleasurable primary emotions, namely PLAY, CARE, SEEKING, and LUST. In addition, there are three aversive primary emotions, which can be subdivided into FEAR and ANGER and a PANIC/GRIEF or SADNESS system. In an evolutionary sense, each primary emotion has the adaptive function to inform the organism of a current homeostatic need (Solms, 2021). In broad strokes, the affective systems can be characterized as the following: (1) PLAY is characterized by fun and play with conspecifics, shared laughter, physical contact, and pleasure with the aim of establishing social contacts and, for example, generating skills in the sense of social competence. (2) CARE is characterized by caring toward the offspring, but also for others. This ensures the basic evolutionary idea of children growing up well and helping each other. (3) SEEKING is shaped by the search for food and choice of partner, and is manifested, for example, in curiosity and problem-solving skills, thus generally giving us the energy to cope with everyday life. The positive primary emotion (4) LUST is characterized by the sexual drive and pleasure gain and thus, from an evolutionary perspective, fulfills the purpose of reproduction for the preservation of the species. The negative primary emotion (5) FEAR expresses itself in the sense of “fight or flight” and has a protective function. The (6) ANGER or RAGE system manifests itself in verbal or physical form and is thus also intended to serve the own survival of others and that of others. The so-called (7) PANIC/GRIEF or SADNESS system is triggered by experiences of separation and can manifest itself in “psychological pain” (Davis et al., 2003; Panksepp, 2011; Montag et al., 2021). The anatomy of the brain is considered to be hierarchically developed and the primary emotions are therefore regarded as *primary processes*, which are located in the developmentally old, subcortically located areas of the brain. Higher up in the hierarchy are the *secondary processes*, such as emotional learning, and finally the neocortically located *tertiary processes*, which relate to complex cognitive components (Panksepp and Watt, 2011).

Previous research regarding mental health and primary emotions has found clinically relevant associations regarding a variety of psychopathological phenomena including depression, somatization, anxiety disorders, personality disorders, obsessive-compulsive disorders, and autism (see Brienza et al., 2023 for a detailed review). Eating disorders show very high comorbidity rates with various psychiatric disorders, these are highest for anxiety and mood-related disorders (prevalence rates up to over 50%). There are also high correlations with disorders related to substance abuse, as well as post-traumatic stress syndrome, personality disorders, autism, and other medical comorbidities (Hambleton et al., 2022). Due to these multiple very high comorbidity rates, we expect similar patterns of association between eating disorders and primary emotions.

To our knowledge, there is no previous work, which has specifically addressed the association of eating disorder symptoms with primary emotions. Empirical research has proposed that ED symptoms are related to dysfunctional affect regulation capacities (Dingemans et al., 2017; Henderson et al., 2019; Prefit et al., 2019; Trompeter et al., 2021). Within the framework of primary emotions this might translate to generally increased negative and decreased positive affects.

In connection to the role of affect regulation, EDs symptoms have been repeatedly associated with insecure attachment internalizations (Zachrisson and Skårderud, 2010).

Adult attachment patterns can be broadly divided into a secure attachment style and an insecure attachment style, the latter of which can manifest as attachment anxiety or attachment avoidance (Bowlby, 1988). Attachment anxiety is characterized by hyperactivation of the attachment system, whereby nagging emotional responses are amplified by arousing pathways, contributing to an upregulation of emotions (Shaver and Mikulincer, 2002). This later manifests in heightened fears of loss and separation. In contrast, attachment avoidance is associated with deactivation and manifests itself in suppression of affective reactions in close relationships (Fraley and Shaver, 2000; Shaver and Mikulincer, 2002). In line with the previously mentioned theory of the hierarchical organization of the brain, attachment patterns in terms of emotional learning can be classified as part of the secondary processes, which are therefore downstream of the primary emotions as primary processes (Panksepp and Watt, 2011).

Clinically, Mikulincer and Shaver (2007) described the connection of attachment insecurity with several mental disorders in numerous studies, ranging from mild impairments to severe mental disorders. With regard to EDs, Zachrisson and Skårderud (2010) observed higher prevalence rates of insecure attachment among eating disordered patients. Along these lines, Tasca and Balfour (2014) report increased levels of attachment insecurity among eating disordered patients, as well as an association of attachment anxiety and eating disorder severity. To this end, mediation models by Tasca et al. (2009) found partial mediation effects through emotional reactivity on the relationship between attachment insecurity and eating disorder symptomatology, among other factors. Furthermore, Klein et al. (2022) demonstrated associations between attachment anxiety and eating disorder symptoms. This association was fully mediated by personality functioning in a path analysis.

With regard to traumatic experiences, there are also strong associations with both insecure attachment and primary emotions, especially the negative emotion SADNESS. Thus, on the one hand, attachment in the sense of psychosocial learning, as well as biological conditions, is already significantly influenced by trauma in earliest childhood (Lahousen et al., 2019). Fuchshuber et al. (2018) were able to demonstrate a mediating effect, including increased SADNESS, between childhood trauma and depression. In a further example, Mariani et al. (2021) showed the primary emotion SADNESS as a relevant risk factor with regard to emotion regulation in the wake of the COVID-19 pandemic as a traumatic event.

Together the internalized adult attachment representations might work like a gateway in regard to affective traits. Similar to the relationship between primary caretaker and infant, attachment internalizations act as affect regulating corrective in adulthood. Along these lines, insecure working models of self and others might aggravate negative and hamper positive affective traits.

### 1.1 Study aims

**FIGURE 1:**
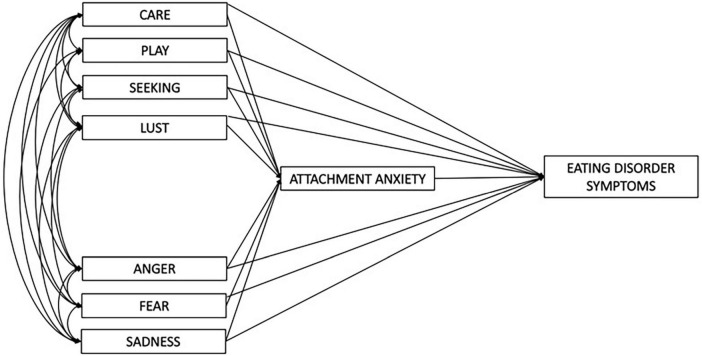
Draft of the path model including all seven primary emotions.

In line with previous findings, we expect (1) that relevant associations of eating disorder symptoms with both primary emotions and attachment patterns can be demonstrated. (2) Path analysis will be applied in order to investigate whether insecure attachment in terms of secondary processes has mediates the association between primary emotions as primary processes and eating disorder symptoms as outlined in Figure 1. The path analytic technique will (3) enable the estimation of potential independent regression weights and indirect effects.

## 2 Materials and methods

### 2.1 Sample and ethics statement

All study participants were recruited via social platforms (Facebook, Instagram, etc.), as well as the mail distribution list of the University of Graz (student.umfrage). Subjects were then encouraged to complete an online self-assessment survey, first requiring informed consent. After collecting some sociodemographic data, including gender, age and existing psychiatric conditions, various questionnaires on primary emotions, attachment, and psychopathology then were conducted. A total of 921 adult subjects who completed the online survey with plausible and coherent answers were included in the analysis. All underage subjects, incomplete questionnaires and those containing contradictory or nonsensical answers were disqualified prior to evaluation. The study was conducted in accordance with the Declaration of Helsinki and with approval obtained from the Ethics Committee of the Karl-Franzens University of Graz in August 2022. Data collection took place from September 2022 to January 2023.

### 2.2 Psychometric assessment

To assess *eating disorder symptoms*, the eight-item *Eating Disorder Examination – Questionnaire 8 (EDE-Q8)* was used, which includes the four subscales Restraint, Eating, Weight, and Shape Concern. In order to make the corresponding questions more comprehensible, some example items are given below: *Restraint* refers to restricted eating behavior or dietary rules [e.g., “Have you tried to exclude from your diet any foods that you like in order to influence your shape or weight (whether or not you have succeeded)?”]. *Eating Concern* refers to the preoccupation with food or the fear of losing control [e.g., “Has thinking about food, eating or calories made it very difficult to concentrate on things you are interested in (for example, working, following a conversation, or reading)?”], *Weight Concern* refers to the focus on weight in general or the wish to lose weight (e.g., “Have you had a strong desire to lose weight?”), and *Shape Concern* refers to the feeling of being obese or, for example, the worries about undressing (e.g., “Have you felt fat?”). The questionnaire refers to frequency and intensity over the past 28 days and contains items adapted from the symptom questionnaire according to the DSM-5 (Hilbert and Tuschen-Caffier, 2016). Reliability in the present non-clinical sample proved to be excellent for the total score (α = 0.93), as well as ranging from acceptable to excellent for all subscales (0.73 ≤ Cronbach’s α ≤ 0.90; Blanz, 2015). This internal consistency is comparable to the reliability values of clinical samples (overall score: Cronbach’s α = 0.97; Hilbert and Tuschen-Caffier, 2016).

The *Brief – Affective Neuroscience Personality Scales – German Version including a LUST Scale (B-ANPS-GL)*, newly validated by Fuchshuber et al. (2023) and comprising a total of 38 items, was used to elicit *primary emotions*. This is based on the “Affective Neuroscience Personality Scales” (ANPS) according to Davis et al. (2003) and represents an abridged version for the survey of the primary emotions CARE, SEEKING, PLAY, as well as FEAR, ANGER and SADNESS and the supplemented seventh primary emotion LUST in German. Accordingly, SADNESS, for example, is represented by items such as “I often feel lonely” or “I often feel sad” or in reverse questioning for their evaluation. These are asked by four to six items each using a five-point Likert scale (1 = “strongly disagree” to 5 = “strongly agree”) and internal consistencies were found to be adequate to good (α = 0.69−0.85) according to Blanz (2015).

The short *questionnaire Experiences in Close Relationships – Revised (ECR-RD8)* was used to assess *attachment insecurity* with the subscales attachment anxiety and attachment avoidance by four items each. The ECR-RD8 evaluates attachment-related expectations and cognitions in relation to partnerships on a seven-point Likert scale (1 = “strongly disagree” to 7 = “strongly agree”). Attachment anxiety thus refers to fear of loss and is depicted with items such as “I worry that romantic partners won’t care about me as much as I care about them.” Attachment avoidance refers to the problems of engaging in a close relationship, an example item would be “It’s easy for me to be affectionate with my partner.” The inventory showed good internal consistencies (α = 0.78–0.85; Ehrenthal et al., 2021).

### 2.3 Data analysis

Using IBM SPSS Statistics 29, all descriptive data, as well as Pearson correlations were output, with two-sided testing of all *p*-values. For this purpose, *p* < 0.01 was specified to counteract alpha error accumulation. The path analysis was estimated using IBM SPSS Amos 28 Graphics and the mediator of attachment anxiety that has the higher association with eating disorder pathology according to zero order correlations is used. After the original model was fit by a pruning strategy that removed all non-significant paths (*p* > 0.05), the goodness of fit was assessed by maximum likelihood estimation. To test for mediation and indirect effects, bootstrap analysis was performed with a bias-corrected confidence interval of 95% and 2,000 bootstrap samples (Cheung and Lau, 2007). The following fit indices were considered for excellent model fit: Root Mean Square Error of Approximation (RMSEA) ≤0.08, Tucker-Lewis Index (TLI), and Comparative Fit Index (CFI) each ≥0.90, and χ^2^/df < 3 (Kline, 2015).

## 3 Results

A total of 1,566 subjects participated in the study collected online via LimeSurvey. A total of 921 who completed the entire test battery and provided coherent and comprehensible responses were included in the data analysis. Furthermore, only those who agreed to the informed consent form at the beginning of the study were included. The sample consisted of 92.6% subjects of German-speaking origin from the countries Germany, Austria, and Switzerland. Study participants ranged in age from 18 to 73 years (*M* = 27.69, SD = 9.61), 69.9% assigned themselves to the female gender, and 85.9% reported a high school diploma. The mean BMI was 23.42 (SD = 4.42), and 13.9% reported a diagnosed psychiatric disorder. Supplementary descriptive data can be found in Table 1.

**TABLE 1:** Sample description with proportions and mean values of the variables of interest.

		*M* (SD)			%
Descriptives	Age	27.69 (9.61)	Sex	Female	69.9
	BMI	23.42 (4.42)		Male	27.8
				Divers	2.3
ECR-RD8	Global	23.79 (8.80)	Sexual orientation	Heterosexual	75.2
	Anxiety	13.00 (5.91)		Homosexual	3.5
	Avoidance	10.79 (5.38)		Others	21.3
			Educational status	High school diploma	85.9
EDE-Q8	Global	12.11 (11.95)	Relationship status	Single	48.5
	Restraint	2.71 (3.46)		Married	11.8
	Weight concern	3.73 (3.79)		Current relationship	36.5
	Eating concern	1.65 (2.43)	Origin	Germanophone	92.6
	Shape concern	4.02 (3.71)	Diagnosed mental disorder		13.9

B-ANPS-GL, Brief – Affective Neuroscience Personality Scales – German Version including a LUST Scale; ECR-RD8, Experiences in Close Relationship – Revised; EDE-Q8, Eating Disorder Examination Questionnaire 8.

### 3.1 Correlations

Pearson correlations showed predominantly low correlations (according to Cohen, 1988) between the positive primary emotions CARE, PLAY, and LUST and global eating disorder symptoms (*r* = −0.10 to −0.22, all *p* < 0.01), and stronger correlations with the subscales Eating Concern and Shape Concern (*r* = −0.09 to −0.24, all *p* < 0.01). The primary emotion SEEKING showed only marginal correlations with the Eating Concern subscale (*r* = 0.08, *p* < 0.05).

The two negative primary emotions SADNESS and FEAR showed moderate correlations with the Shape Concern subscale (*r* = 0.29, *p* < 0.01), and small to moderate correlations with the total score, as well as all other subscales (*r* = 0.14 to 0.28, all *p* < 0.01). ANGER showed generally small correlations (*r* = 0.11–0.13, all *p* < 0.01) with eating disorder symptoms, as well as a marginal correlation with the Restraint subscale (*r* = 0.08, *p* < 0.05). All positive primary emotions showed negative correlations (*r* = −0.19 to −0.25) and all negatives showed positive correlations with attachment insecurity (*r* = 0.25–0.44, all *p* < 0.01). Eating pathology was also more strongly related to attachment anxiety across all subscales (*r* = 0.14–0.24, all *p* < 0.01). All correlations can be found in Table 2.

**TABLE 2:** Descriptive statistics and zero-order correlations for indicator variables.

	1	2	3	4	5	6	7	8	9	10	11	12	13	14	15
1. PLAY	–														
2. CARE	0.44**	–													
3. SEEKING	0.29**	0.21**	–												
4. LUST	0.38**	0.40**	0.19**	–											
5. ANGER	-0.09**	-0.15**	-0.11**	-0.08*	–										
6. FEAR	-0.16**	0.02	-0.06	-0.28**	0.26**	–									
7. SADNESS	-0.24**	-0.06	-0.10**	-0.35**	0.34**	0.68**	–								
8. Attachment insecurity	-0.25**	-0.27**	-0.19**	-0.36**	0.25**	0.24**	0.44**	–							
9. Attachment anxiety	-0.09**	0.02	-0.08*	-0.18**	0.17**	0.29**	0.42**	0.75**	–						
10. Attachment avoidance	-0.32**	-0.46**	-0.22**	-0.40**	0.22**	0.07*	0.26**	0.80**	0.21**	–					
11. Global eating pathology	-0.14**	-0.10**	-0.04	-0.22**	0.12**	0.27**	0.26**	0.20**	0.22**	0.08*	–				
12. Restraint	-0.10**	-0.08*	0.01	-0.13**	0.07*	0.18**	0.14**	0.13**	0.14**	0.06	0.85**	–			
13. Weight concern	-0.11**	-0.08*	-0.03	-0.19**	0.11**	0.23**	0.23**	0.16**	0.19**	0.06	0.94**	0.69**	–		
14. Eating concern	-0.16**	-0.10**	-0.08*	-0.21**	0.11**	0.26**	0.28**	0.23**	0.24**	0.11**	0.85**	0.71**	0.72**	–	
15. Shape concern	-0.16**	-0.09**	-0.05	-0.24**	0.13**	0.29**	0.29**	0.21**	0.23**	0.09**	0.92**	0.63**	0.89**	0.70**	–
16. Age	-0.13**	-0.09**	-0.06	0.06	0.01	-0.26**	-0.19**	-0.03	-0.12**	0.09**	-0.06	-0.02	-0.04	-0.14**	-0.06
17. BMI	-0.01	-0.03	-0.02	0.05	0.08*	-0.08*	-0.04	0.06	0.01	0.09**	0.23**	0.11**	0.28**	0.10**	0.28**
Cronbach’s α	0.76	0.69	0.72	0.79	0.80	0.84	0.85	0.78	0.79	0.85	0.93	0.90	0.87	0.73	0.87

*n* = 921; ** *p* < 0.01; * *p* < 0.05. Sex was coded as 1, female; 2, male; 3, diverse.

### 3.2 Path analysis

Within a path model, the seven primary emotions were correlated with each other and assessed for effects on eating disorder symptoms. Based on the zero-order correlation matrix, only attachment anxiety was included as a potential mediator. Before trimming, the model showed the following model fit: RMSEA = 0.67 (90% CI: 0.04, 0.10), TLI = 0.96, CFI = 1.00, χ^2^ = 15.27 (*p* = 0.002, df = 3). Good to acceptable fit indices resulted after trimming out all non-significant pathways: RMSEA = 0.06 (90% CI: 0.03, 0.08); TLI = 0.97 and CFI = 1.00, χ^2^ = 22.53 (*p* = 0.001, df = 6). In relation to the model fit, the RMSEA of 0.06 in this analysis according to Hu and Bentler (1999) is barely good, according to MacCallum et al. (1996) it is to be classified as moderate.

### 3.3 Direct and indirect effects

At the local level, eating disorder symptoms were directly associated with LUST (β = −0.13, *p* < 0.001), and FEAR (β = 0.17, *p* < 0.001). Furthermore, attachment anxiety was significantly linked to eating pathology (β = 0.14, *p* < 0.01). SADNESS showed a substantial effect on attachment anxiety (β = 0.41, *p* < 0.01).

Broken down into the various subscales, there are direct effects of LUST on Restraint (β = −0.07), Weight (β = −0.12), Eating (β = −0.12, all *p* < 0.001) and Shape Concern (β = −0.15, *p* < 0.05). FEAR was directly associated with Restraint (β = 0.12, *p* < 0.05), Weight (β = 0.14), Eating (β = 0.10), and Shape Concern (β = 0.19, all *p* < 0.001). SADNESS was directly associated with Eating Concern (β = 0.08, *p* < 0.05) and Attachment Anxiety (β = 0.40, *p* < 0.001).

Bootstrapping analysis indicated that the association between SADNESS and eating disorder symptoms was fully mediated by attachment anxiety (β = 0.06, CI = 0.03, 0.09, *p* = 0.001).

The relationship between SADNESS and the Shape (β = 0.06, *p* = 0.001) and Weight Concern (β = 0.05, *p* = 0.001) subscales was also fully mediated by attachment anxiety.

**FIGURE 2:**
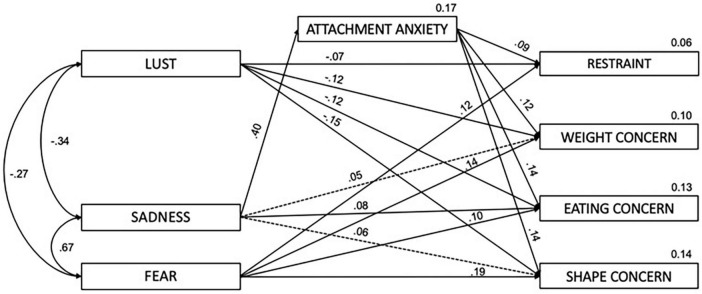
Mediated path analysis, indirect effects in dashed lines; controlled for sex and age.

The final model is illustrated in Figure 2. In sum, the model was able to explain a total of 17% of the variance in attachment anxiety and in regard to the eating disorder subscales 6% for Restraint, 13% for Eating, 10% for Weight, and 14% for Shape Concern.

## 4 Discussion

This study addressed the association of primary emotions and attachment patterns on eating disordered symptoms. Overall, LUST and FEAR were directly linked to eating pathology, while the association between SADNESS and weight and shape concern was fully mediated by attachment anxiety. While the observed associations were generally small, the results are largely consistent with previous literature.

(1) As hypothesized, positive associations were found between global eating pathology and the negative primary emotions ANGER, SADNESS, and, most strongly, FEAR. Negative associations were observed with the positive emotions LUST and, to a lesser extent, PLAY. CARE showed only marginal associations, and SEEKING exhibited no associations. However, the associations with ANGER emerging from this study were very low in magnitude and, according to Cohen (1988), should be classified as weak. Especially findings of decreased LUST relating to eating disorder symptoms resonate well with previous literature linking sexual dysfunction with bulimia nervosa and anorexia nervosa (Wiederman, 1996; Caparrotta and Ghaffari, 2006; Castellini et al., 2016, 2020).
(2) The associations between eating pathology and attachment insecurity, were stronger for global ED pathology, as well as across all subscales for attachment anxiety than with attachment avoidance. Accordingly, these tendencies toward attachment anxiety were also found, for example, in Tasca and Balfour (2014) and Klein et al. (2022).
(3) In the core of the study, the path model suggested, independent effects of primary emotion LUST, FEAR, and attachment anxiety, contributing to eating disorder symptoms across all subscales. In the mediation analysis, the association between the negative primary emotion SADNESS and weight and shape concern were fully mediated by attachment anxiety.

The observed mediation appears plausible, as especially attachment anxiety is characterized by agonizing emotional upregulation and is associated with increased fear of separation and loss (Fraley and Shaver, 2000; Shaver and Mikulincer, 2002; Montag et al., 2021). As mentioned above, there is a strong association of various traumas with SADNESS and attachment insecurity, which is also essential in the development of a range of psychopathologies (Fuchshuber et al., 2018, 2019a,b; Lahousen et al., 2019; Mariani et al., 2021). Thus, the connection found between this complex interaction should be examined in more detail in further studies, e.g., with a focus on different kinds of traumatic experiences.

Since eating disorders have an extremely complex psychopathology with many etiological determinants, a degradation of LUST is of high interest. On the one hand, early childhood experiences play a significant role, with the topic of trauma again coming into focus. On the other hand, biological factors, but also psychological factors such as self-perception and disgust, play a fundamental role (Pinheiro et al., 2010; Castellini et al., 2016). Hence, this connection might also be considered from a psychoanalytic perspective in future studies.

Overall, the correlations and interactions found are congruent with existing research and despite a non-clinical sample and the limited informative value of the BMI, extremely low values of ≤18.5, for example, which were found in 5.4% of the sample, could possibly indicate an existing psychopathology.

### 4.1 Limitations

This study also includes some limitations that should be noted: the study relies solely on self-report measures and lacks interviews or structured diagnostic criteria specific to different disorders within the eating disorder spectrum. This type of survey requires a high degree of self-reflection and honesty. In addition, answers could be given in the sense of social desirability. Especially with LUST, Davis and Panksepp (2011) feared a possible lack of frankness. Another limitation is that this study has a cross-sectional design, not allowing for causal inferences. While a longitudinal design would be necessary for establishing causality, this study contributes additional insight into eating disorder pathology. It should also be noted that while the sample is drawn from the general population, it includes a rather skewed level of education with 85.9% of participants having at least an university entrance qualification, and is restricted to German-speaking countries (92.6% German-speaking country of origin), thus limiting generalizability.

## 5 Conclusion

Overall, the associations observed in this study may pose an interesting contribution to the understanding of possible etiological factors in eating disorders. Due to the fact that “social species’ brains do not exist in isolation” (Holmes and Slade, 2019, p. 318) combining the approaches of primary affective traits in connection with attachment theory might provide additional insight and new food for thought for the therapeutic work with ED patients.

## Data availability statement

The raw data supporting the conclusions of this article will be made available by the authors, without undue reservation.

## Ethics statement

The studies involving humans were approved by the Ethics Committee of the University of Graz. The studies were conducted in accordance with the local legislation and institutional requirements. The participants provided their written informed consent to participate in this study.

## Author contributions

LR: Writing – original draft. JF: Writing – review & editing. TP: Writing – review & editing. DA: Writing – review & editing. BS: Writing – review & editing. AS: Writing – review & editing. H-FU: Writing – review & editing.
